# “Eventually, I Admitted, ‘I Cannot Do This Alone’”: Exploring Experiences of Suicidality and Help-Seeking Drivers Among Australian Men

**DOI:** 10.3389/fsoc.2021.727069

**Published:** 2021-10-01

**Authors:** Zac E. Seidler, Michael J. Wilson, John L. Oliffe, David Kealy, Nicholas Toogood, John S. Ogrodniczuk, Simon M. Rice

**Affiliations:** ^1^ Orygen, Parkville, VIC, Australia; ^2^ Centre for Youth Mental Health, The University of Melbourne, Melbourne, VIC, Australia; ^3^ Movember, Melbourne, VIC, Australia; ^4^ School of Nursing, University of British Columbia, Vancouver, BC, Canada; ^5^ Department of Nursing, The University of Melbourne, Melbourne, VIC, Australia; ^6^ Department of Psychiatry, University of British Columbia, Vancouver, BC, Canada

**Keywords:** suicide, masculinity, help-seeking, men’s mental health, gender

## Abstract

While research has explored the intersection between masculinities and men’s experiences of suicidality, comparatively little attention has been paid to the stories surrounding suicidal men’s decision to seek help. The ways in which men experiencing suicidal thoughts and behaviors embody masculinities alongside their enlistment of mental health services remains largely unknown. The present study explored 262 Australian men’s stories surrounding the impetus for help-seeking for suicidal thoughts and behaviors. The sample comprised men ranging in age from 17 to 74 years (*M* = 40.99; SD = 15.92 years), with most participants residing in a metropolitan area (55.3%), employed full time (43.1%), non-indigenous (95.4%) and heterosexual (73.7%). Participants elaborated on their reasons for help-seeking *via* an open-text qualitative survey, delivered as part of a larger study exploring help-seeking experiences of Australian men. Thematic analysis of responses generated four themes highlighting the diversity of experience across men, with some highlighting impacts of emasculating early trauma(s) on their suicidality, while others reflected an impulsiveness tied to situational stressors that fractured their masculine identity (e.g., relationship breakdown; job loss). Many men had epiphanies as they reached the limits of their self-reliance and came to terms with their need for help. As their suicidality was witnessed by—and began to impact—those around them, the sight of their previously masked pain by others often facilitated their help-seeking journey. The present findings underscore the complex and multifactorial role of masculinities in men’s suicidality and their paths to help-seeking. Important inroads for future public mental health promotion efforts are discussed, in terms of leveraging self-reliant and caring masculinities in helping men to develop healthy coping in the context of suicidality.

## 1 Introduction

Addressing high and rising male suicide rates is an urgent public health issue. Research efforts contributing to this work often manifest as intersections of sociology and mental illness studies to advance understandings of male suicidality (Cleary, 2019). Men constitute up to 80% of the one-million suicide deaths recorded each year worldwide (WHO, 2018), and consistently make up three-quarters of all suicide deaths in Australia (ABS, 2020). Improving understanding of the conditions by which men develop, cope with and respond to suicidality will inform targeted interventions for men.

The role of socialised masculinity has long been threaded through commentaries and narratives explaining male suicide, in an effort to understand the gendered dimensions of this phenomenon. This understanding developed from an early, largely reductionist perspective proposed by Durkheim (1951) that considered male suicide through broad sociocultural patterns. Yet this perspective was thought to undermine the utility of individual intervention, paving the way for more recent, nuanced and largely qualitative exploration of men’s unique contextual pathways to suicidality (e.g., Richardson et al., 2021a). Consistent across more recent efforts to understand suicide in men is an overt focus on the role of men’s gender socialisation; that is, the social and developmental experiences that provide a blueprint for each man’s expression of their masculinity (Addis and Mahalik, 2003). The archetype of masculinity often discussed in relation to men’s mental health is referred to as “traditional masculinity,” which carries underpinning tenets of strict stoicism, self-reliance and strength. These factors are often framed as driving forces in men’s challenges dealing with distress and undermining their willingness to seek help (Seidler et al., 2016; Pirkis et al., 2017).

Since Durkheim’s (1951) work, the rise of gender relations theory through Connell and Messerschmidt, (2005) masculinities framework has permeated our understanding of how men experience and exhibit their gender. In this context, gender relations theory explores the ways in which this idealised, oft-stereotyped “traditional” masculinity of the straight, white, middle-class and able-bodied man is one of many possible enactments of manhood that intersect and diversify based on geographical, cultural, racial and sexual factors. In extending past sex-differences research to exploring within-men differences by seeking personalised accounts, current empirical endeavors aim to dispel often harmful stereotypes that cast men as a singular category and their suicidality as being underpinned by universal factors like economic hardship (Qin et al., 2000).

In an effort to understand male-specific risk factors for suicidality, and to ensure mental health promotion interventions are attuned to male populations, masculine socialisation has often helped contextualise these issues. Foremost among these risk factors are alcohol and substance overuse, being single, and a depression diagnosis (Richardson et al., 2021b). Underpinning many of these risks are the situations that give rise to distressing periods of transition for many men, including unemployment, financial distress, and relationship breakdown (Clapperton et al., 2019; Cunningham et al., 2021). The emergence of these risk factors also accords with broader modelling of the conditions under which suicidal ideation arises, where Nguyen et al. (2021) identified high degrees of social connectedness and perceived availability of help-related sources and information as necessary for the prevention of suicidal thoughts. Tracing these risks back to gender socialisation implicates the often maladaptive, yet masculinity-sanctioned, coping strategies that allow men to conceal and compensate for their distress and, in turn, accrue “masculine capital” which confers perceived strength and competency (Player et al., 2015). Indeed, extensive literature has examined associations between adherence to traditional masculine role norms (particularly self-reliance) and greater propensity for suicide risk (e.g., Pirkis et al., 2017; Feigelman et al., 2021). Much of this research has been conducted among Australian men, highlighting the particular salience of Australian traditional masculinities sanctioning independence, stoicism and strength when considering risk of suicidal thinking (Pirkis et al., 2017), suicide attempt (Player et al., 2015) or help-seeking experiences in relation to depression and suicidality (River, 2018; Seidler et al., 2018).

One proposed explanation for this association concerns the speed of progression from initial stressor, to suicide ideation, to suicidal behaviour believed to occur in many men (Schrijvers et al., 2012). Impulsivity, poor problem-solving capacity, and a propensity for risk-taking behaviour can be characteristic of depression and suicidality in men (Player et al., 2015; Rice et al., 2019). As Hunt et al. (2017) highlight, some men’s desire to alleviate their pain and distress outweighs their resources for coping with this hardship, resulting in death by suicide. The higher likelihood of death following a suicide attempt in men compared to women has also been grounded in masculinity, with access to and/or the greater propensity to choose lethal means thought to interact with that risk-taking and impulsivity (e.g., hanging, firearms; Roy et al., 2013). As such, it is clear that our understanding of the interplay between masculine socialisation and the onset and enactment of suicidality in men has grown in recent years. Comparatively little research has examined men’s help-seeking for suicidality, and the road to recovery, through a masculinities lens.

Men’s reticence to seek help in the context of suicidality has long been chronicled in sex differences research. Yet more pertinent to modern scholarship surrounding men’s help-seeking for suicidality is research reporting increased rates of mental health service access among men (Harris et al., 2015). Most concerning, in the year prior to suicide, 91% of men had some form of healthcare contact, and up to two-thirds of men had contact specifically with mental health services (Schaffer et al., 2016; Appelby et al., 2021). This indicates a need for research focused on suicidal men’s help-seeking. This will help to ensure that when men arrive at services, practitioners are equipped to respond to what is known to be a short window of opportunity for effective intervention (River, 2018).

Whilst men’s help-seeking in the context of depression has received significant attention (Johnson et al., 2012; Seidler et al., 2016), relatively little research has investigated men’s help-seeking for suicidality. Exceptions include River’s (2018) suicidality work contrasting men seeking help to preserve masculine capital, and those who suggested they had “nothing to lose,” with those other men steadfastly avoiding services. Beyond this, focus-group data has highlighted how suicidal men appear to engage in a complex process of negotiation with ideas and methods of help-seeking whilst avoiding compromising their masculinity and a concurrent loss of agency (Keohane and Richardson, 2018). Additionally, Ridge et al. (2021) examined how at-risk men progress towards, and then ultimately avoid suicide, finding that social connections and masculine notions of “fighting back” against distress can prove instrumental in men’s decisions to enact positive change. Taken together, across the cited studies it is clear that masculinities can shape men’s risk for suicidality, downward trajectories of distress, the nature of their attempts, and their approaches to help-seeking.

The interaction between masculinities, suicidality and help-seeking therefore requires further investigation, such that the details of men’s help-seeking are not neglected amidst a familiar narrative predicated on the belief that “men don’t seek help.” Whilst barriers to men’s mental health help-seeking have received much attention (e.g., Lynch et al., 2018; Seidler et al., 2019), further work is required to elucidate facilitators for help-seeking among men who have experienced suicidality. This will develop the existent male suicide literature that explores the road to suicidality, by seeking out the narrative tied to the “critical moment” (Giddens, 1991) of survival; that is, what happened that kept them alive. Research examining the factors that underpin the decision of suicidal men to take their distress to professional services is therefore critical if we are to equip services to respond effectively to men’s needs with masculinities in mind.

As such, an important next step for the field is to gain additional understanding of the paths from risk factors to actual experiences of suicidality as reported by help-seeking men. The aim of the current study and article is to gain greater depth of understanding concerning the drivers of help-seeking among suicidal men. The current study also aims to explore the ways in which masculinities intertwine with suicidal men’s help-seeking.

## 2 Methods

### 2.1 Design

The present study employed a cross-sectional design, involving open-ended qualitative survey responses. To gain a “wide-angle picture” (e.g., Toerien and Wilkinson, 2004, p. 89) of therapist perspectives, open-text survey responses represented a viable avenue to garner insights from Australian respondents from a broad array of demographics. A qualitative study design was also used to enable access to suicidal men’s life stories and experiences surrounding their help-seeking for suicidality (Braun et al., 2020). Open-text online surveys exploring men’s help-seeking for suicidality provided an avenue to collect narrative data of suicidal men’s own words concerning their suicidality and help-seeking; while ease of data collection allows greater numbers of men to be included, building on previous research. Applying online surveys as a qualitative research tool has grown in scope in recent years (Braun et al., 2020). This method can encourage participants to anonymously produce their own language and meaning to reflect their experiences (Frith, 2000; Braun and Clarke, 2013). Offering men the opportunity to describe their stories of suicidality and help-seeking in as much depth as they find comfortable, is also of value given known advantages afforded by granting men anonymity, confidentiality and brevity when disclosing their experiences of distress (Tyler and Williams, 2014; Hanna and Gough, 2016; Driolli-Phillips et al., 2021).

### 2.2 Procedure

Between March and May 2020, Australian men aged 16 and over (*N* = 2,009) were invited to complete a ∼15-min survey about their experiences in mental health treatment (i.e., talk therapy or counselling). Survey advertisements were delivered *via* the Facebook page of Movember, the world’s leading men’s health charity. The following advertisement text was used: “*Have you ever had counselling or therapy? If you can spare 15 min, we’d love to hear from you so we can improve therapy for men.*” Individuals who clicked on the advertisements were presented with a plain language statement and consent form, and were required to provide informed consent (*via* a yes/no survey item) prior to progressing to the body of the survey. To compensate for their time, participants were given the option to provide their email address to enter a prize draw for one of fifty $100 gift vouchers.

### 2.3 Data Collection

Participants in the broader survey responded to a mix of quantitative and qualitative survey items, alongside several standardised measures of mental illness. Responses to two survey questions were used to derive the analysis sample for this study. The items applied in this survey were developed in consultation with the author group, who represent leading experts in masculinities, men’s mental health and suicidality.

Participants initially responded to a purpose-designed item which categorically assessed the main reason they had sought help in their most recent experience of therapy: “*Thinking about your most recent experience of therapy, what was the main reason you sought help?*” A range of response options reflecting different disorders (e.g., anxiety; depression; psychosis) and situational concerns (e.g., work-related stress; relationship issues) were provided, including an option designed to delineate those men who specifically sought help for suicidality: “*suicidal thoughts/behaviour*”.

Following this, participants were asked to elaborate on this in their own words, *via* an open-ended qualitative question: “*More broadly, in your own words, what led you to seek help from a therapist?*”

### 2.4 Data Analysis

The present study applied thematic analysis of open-ended qualitative survey responses (Braun and Clarke, 2006). From the outset, the data was approached with a masculinities lens (Connell and Messerschmidt, 2005), where men are understood to embody various interconnected characteristics that are context- and identity-dependent. Importantly, the line of questioning did not mention masculinity and therefore this lens resided with the researchers. Throughout the analysis, we sought to bring to light and thread evidence of the ways in which men negotiate masculinities alongside their experiences of help-seeking for suicidality.

To prepare for analysis, those responses of participants who categorically indicated they sought help for suicidality were downloaded to a spreadsheet. A six-stage process of thematic analysis was then followed, according to Braun and Clarke (2006), with special consideration given to this being thematic analysis of open survey data as per Braun et al. (2020).

Responses were initially reviewed in-depth by two authors (ZS and MW), with basic concepts coded independently. Codes were reviewed for consistency in consultation with a third author (NT), and any inconsistencies were amended and agreed upon according to group consensus among the three authors. Labels for initial codes were then adjusted for ease of interpretation and codes with overlapping concepts were combined. For example, the codes “painful separation” and “relationship breakdown” were combined into one entry reflecting relationship breakdown. These codes were then grouped based on thematic similarity leading to the generation of preliminary themes. For example, often respondents described a job loss, financial difficulties or relationship breakdown as the primary trigger in their distress. These factors were initially coded separately, but were subsequently unified into a theme called situational suicidality detailing the relatively short trajectories into suicidality described by men who had experienced these largely situational, psychosocial stressors.

The preliminary themes were then reviewed and revised in order to achieve a complete picture of the data, where some smaller themes were subsumed into larger themes. For example, the situational suicidality theme was broadened in scope to the final theme which better captured the sense of cumulative and cascading losses evident in men’s suicidality, one component of which was often a situational factor. These themes were then reviewed collaboratively by all authors to ensure they best captured the essence of the data collected. Analytic and reflexive notes and memos were developed alongside coding and theme development, which were subsequently discussed and refined collaboratively in team meetings among the author group, in order to reduce individual researcher bias and guide the analysis. Finally, meetings among the author group represented the platform for the achievement of consensus for the final themes and illustrative quotes, presented in the results section below.

## 3 Results

In total, 262 participants selected “suicidal thoughts/behaviour” as their main reason for seeking help in their most recent experience of help-seeking and were thus included in this study. For this group, their age ranged from 17 to 74 (*M* = 40.99; SD = 15.92). Most participants lived in a metropolitan area (55.3%, *n* = 145), identified as non-Indigenous (95.4%, *n* = 250), and heterosexual (73.7%, *n* = 193). Most participants (61.8%, *n* = 162) reported current employment (the majority of those in full-time employment 69.8%), while 17.6% (*n* = 46) were unemployed. Most participants were partnered or married (50.8%, *n* = 133), 32.8% were single (i.e., never married; *n* = 86), 14.1% identified their relationship status as separated or divorced (*n* = 37), and 2.3% were widowed (*n* = 6). The majority of participants identified as cisgender men (98.9%, *n* = 259), while 2 participants identified as transgender men (0.8%) and one identified their gender as non-binary (0.4%).

Many respondents described the impetus for their help-seeking for suicidality by declaratively listing a complex set of interconnected diagnoses, symptoms, and/or psychosocial stressors, the most common of which were depression, anxiety, trauma and substance misuse. These responses typically lacked any descriptive quality surrounding temporality, severity or triggers. The language used in these short responses often mapped onto medicalised language that may have stemmed from their interaction with mental health services, providing a diagnostic lens through which they could contextualise the breadth and depth of their suffering and reliably describe their distress. For others, this listing lacked more formal diagnostic language, but retained a similar quality of a build-up of a concurrent web of issues that overwhelmed the man and led him on the road to both suicidality, and as a result of this severity of distress, consequent help-seeking.

### 3.1 Thematic Analysis

**FIGURE 1:**
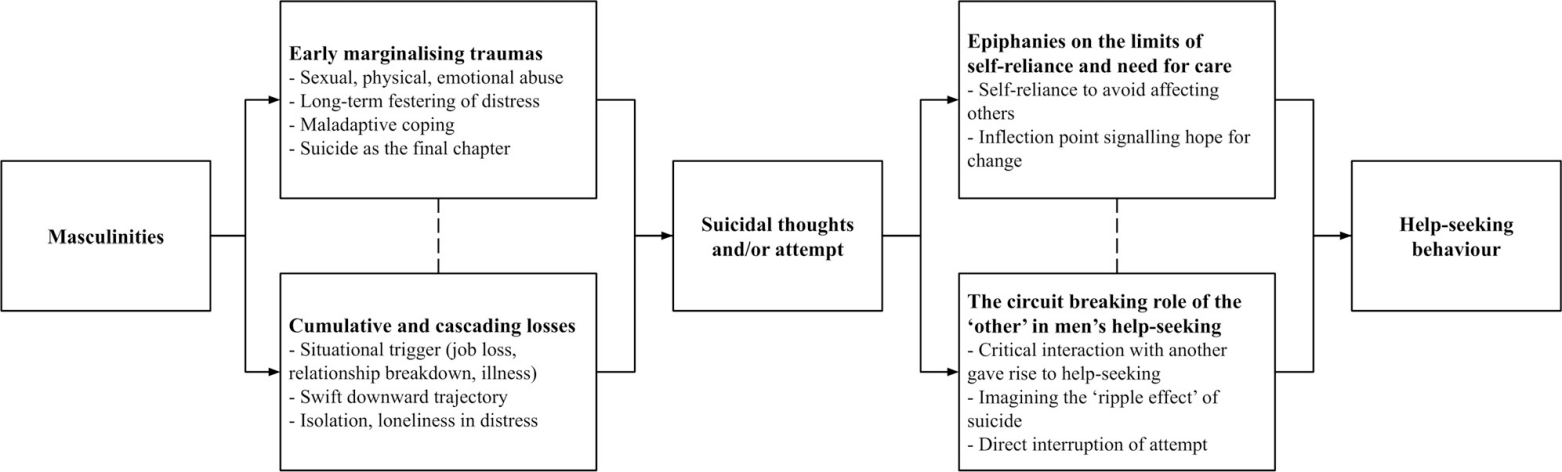
Graphical depiction of themes generated from the data exploring suicidal men’s help-seeking.

Thematic analysis led to the generation of four themes, described in turn below. Figure 1 also provides an overview of the themes generated from the data.

#### 3.1.1 Early Marginalising Traumas

Many respondents provided lengthy, in-depth accounts of the stressors they had experienced throughout their lives, and foremost among these was the experience of early childhood trauma or abuse (e.g., sexual, verbal, emotional or physical). Many men noted with a sense of inevitability that their trajectory towards suicidality began years prior to their help-seeking. Rather than a rapid escalation in distress, initial early-life trauma and developmental ruptures seemed to give way to behaviour that continued to degrade their quality of life. These experiences were often expressed as diminishing men’s sense of control over their lives, and could therefore be considered emasculating early traumas. If a situational trigger for suicidality was described by these men, many linked it back to the core distress from the past; a festering of issues that had gone unresolved for up to decades at a time. It seemed for many of these men that early trauma set the stage for ongoing vulnerabilities and layering of injurious effects, alongside maladaptive concealment of these emasculating traumas.

*To sort out issues from failed relationships, and constant anxiety about surviving from day to day. Lack of sleep due to shitty thoughts from my past circulating non-stop in my head. Have few happy moments. Never truly happy since child abuse at 7 years old. Have planned own funeral* (71-year-old).

Many of these accounts contained hints at the role of traditional masculinities in rendering troublesome coping strategies as the most accessible in times of need, which seemed to fuel cycles of suffering. At times, the influence of traditional masculine norms of toughness, avoidance of vulnerability and emotional restrictiveness were central in their response.

*For a long time I wanted to seek help. My social anxiety, belief that I had to be “tough”, and not knowing if I was bad enough to seek help, meant that I didn’t want anyone to know what was happening* (22-year-old).

Participants who provided lengthy accounts of a lifetime of distress also indicated a fragmented aspect of their identity. Often linked to early trauma in many responses, these men indicated that modern-day stressors seemed again to bring to light their lack of belonging, or an inability to enact a self-determined masculinity given their fundamental lack of self-acceptance. For some men this came in the form of a lack of self-acceptance of their sexuality, whereas for others this was typified by a physical disability. These men’s long-term experiences of destabilising, marginalised masculinities situated their suicidality as a means to take back control and reassert their power over a seemingly out of control life, ridden with traumas that had stripped them of requisite masculine traits of strength or success. Suicidality therefore seemed to emerge as a “realisation” for these men as inevitable; a final chapter in a life defined by chaos and a viable means to protect against any chance of further suffering, but on their own terms.

*I came to the realisation that I had been emotionally abused as a child by my parents, I had issues around identifying my sexuality, which continue today and then I became aware that I had been on the end of a long term bullying relationship in my workplace and I came to the realisation that driving my ute [utility vehicle] into a telegraph pole would help stop the feeling of hopelessness that had become my predominant feeling.* (51-year-old)

For men who reported a suite of stressors across the lifespan, evident in their responses was also the sense that the path leading to suicidality, and that which led to help-seeking, were directly intertwined. That is, while suicide at first loomed large as the only way to alter their life narrative, help-seeking and mental health intervention eventually seemed like an equally viable logical solution or last option to try. For these men, coming to the understanding that their long-running distress and suicidality was not a typical male experience, and did not simply need to be endured, highlights the inflection point where suicidality tipped into help-seeking.

*I’d been struggling with suicidal ideation since I was 13, it took me years to realise this wasn’t “normal,” my entire family has struggled with suicide and as such it was a somewhat normal thing in my family* (18-year-old).

#### 3.1.2 Cumulative and Cascading Losses

Compared to the early and often ongoing trauma experiences relayed in the previous theme, evident in many participants’ responses was the sense that their suicidality was typified by a relatively short and impulsive trajectory from a situational trigger (e.g., job loss, relationship breakdown, violent event, accident or diagnosis with a serious health condition) to extreme distress. It seemed that the identity of many men was bolstered by the security of these domains that was subsequently shattered. As jobs were lost, financial difficulties ensued and relationships fractured, it seemed the cultural capital with which these men felt comfortable to move through the world was stripped away, and swiftly replaced with suicidality.

While not reporting a long history of mental health difficulties like many of the men above, these participants noted how the impact of this acute event appeared to vastly outweigh their own coping resources, making the need for help-seeking more urgent.

*Completely suicidal after loss of job* (63-year-old).

*I hit rock bottom emotionally and financially* (42-year-old).

This sense of “completeness” to men’s suicidality and their resolute language in communicating about their distress, along with the sense that their worlds had come crashing down, was common across responses. Many participants indicated that their suicidality arose out of the fracturing of an internal standard for themselves typically tied to rigid traditional masculine norms of success, protecting and providing, spurred on by the experience of hardship.

*Dealing with the passing of my wife and issues of suicidal thoughts because I could not save her after caring for her for 5 years and now the pressure of holding the family together* (49-year-old).

Additionally, evident in responses was the sense that many men had managed to maintain control and self-reliance in the face of extensive hardship overtime. However, a downward exponential trend into despair and suicidality arose out of an ostensibly unrelated incident, which was described by one man as his “breaking point.”

*I had a number of work related stress issues which led to a number of breakdowns. I had learned to live with chronic depression but after an illness a minor issue led to a full crash and a fairly recent serious suicide attempt resulting in hospitalisation. I have an ongoing mental health program* (66-year-old).

This event was sometimes also coupled with a withdrawal of previously-available social supports, with many men describing feelings of isolation and loneliness which increased suicidality and made formal help-seeking seem more imperative.

*Marriage breakdown, feeling of betrayal, possible loss of job due to alcoholism and violent behaviour, let down by employer, feeling of isolation, peers turned their back… loneliness, suicidal thoughts* (37-year-old).

Many men reported that the experience of suicidality itself was the crisis point needed to make them decide to seek help, where previously many men had ostensibly endured their distress without considering that help might be available or saw their suffering as too mild-moderate to warrant support.

*It wasn’t until I attempted suicide that I was told to go to my GP to get a referral to see a psychologist and psychiatrist (the hospital also organised community psychiatry appointments in the meantime). It was only then that I thought maybe I really did need help and wouldn’t be wasting their time* (22-year-old).

#### 3.1.3 Epiphanies on the Limits of Self-Reliance and Need for Care

Many responses highlighted the varying degrees to which the maladaptive adherence to inflexible masculine codes of self-reliance plays a role in men’s avoidance of help-seeking for suicidality.

Many respondents resorted to innumerable other harmful coping mechanisms before professional help-seeking became a realistic and/or desirable option. The period leading up to presentation was, for many men, characterised by destructive substance abuse, social withdrawal, avoidance of communication, and eventually self-harm, leading one respondent to bluntly state that “*without counselling I’d simply be dead*.” (49-year-old)

*I isolated myself and self medicated with alcohol, whilst hoarding took over my home. Overwhelmed, I began having frequent visualizations of self harm and suicide. Despite being very self-reliant most of my life, this had now become a pattern of self-analysis and self-deprecation. After retrenchment from my job of 15 years, I was forced to confront how serious my mental state had become. Eventually I admitted I cannot do this alone* (53-year-old).

Yet other responses carried more subtle hints at the ways in which early socialisation into a tendency to avoid emotionally-open and vulnerable conversations, even with loved ones, served to exacerbate some men’s self-reliance in propelling them toward suicidality. For these men, even though they knew the various formal and informal gateways to support, the stark reality of their distress intersected with a strict masculine doctrine of independence and strength. This juncture served to illuminate all of the reasons that sharing their experience would be inappropriate, unhelpful or uncomfortable for others. These men often highlighted how the structures and services created to help them often felt misaligned with their own needs and current context.

*I wanted to kill myself because of the situation I was in at work. I didn’t want to talk too much about it to my partner because she gets stressed out about it. Work brings me nothing but negative experiences because I work in the field of construction as an engineer. It’s a male dominated industry full of huge egos who only care about money. I think speaking to a male counsellor about it would cause them to unconsciously put up defences too* (29-year-old).

In their accounts, many men described the point at which they realised “*something had to change*” and “*there was no alternative.*” They could no longer continue in the absence of professional help. As above, for many respondents, the serious reality of their suicidal thoughts or behaviour was the driving factor in exposing the limits of their self-reliance.

*…I was stuck in repeating behaviours and knew that I didn’t have the ability to move beyond them without help* (46-year-old).

*I was mostly just tired of having to talk myself out of it every day. It’s exhausting and I have other things I’d rather be doing* (38-year-old).

Many respondents identified an epiphanous moment where their hope for change acted as the catalyst for their help-seeking. For some men, this was expressed simply as the desire to change—whether in general, or within a specific domain, evident in responses such as “*I wanted to change*” (35-year-old); and “[*I felt*] *trapped in my own thought patterns and that I just couldn’t really continue living that way.*” (26-year-old) For some respondents, the hope for change took the form of a more explicit wish to alleviate their suffering. These responses acknowledged the extent of their distress while also articulating a yearning for its resolution.

*I want to go some way to resolving the feeling of not having a break from my negative feelings and thoughts, and need help doing that besides with medication* (27-year-old).

*I don’t want to want to kill myself* (20-year-old).

Other responses concerned the prospect of managing their mental health challenges. Rather than focusing on the alleviation of suffering, these men expressed aspirations to improve their ability to function despite their challenges.

*I’ve been struggling with mental health issues all my life and I want to become a fully functional adult* (17-year-old).

The commonality within this group of responses is an aspiration for positive change. These respondents imagined a counterfactual circumstance in which their suffering was attenuated and their functioning was enhanced. Quite apart from either a reflexive response to suffering, or the influence of others, these responses leveraged the hope for change as a driver of help-seeking behaviour.

#### 3.1.4 The circuit Breaking Role of the “Other” in Men’s Help-Seeking

As the distress of many men grew and culminated in suicidality and their ability to self-regulate or hide their internal experience faltered, it often spread into the external world. For many men therefore, it was a form of interaction with someone in their social circle that gave rise to an inflection point toward help-seeking; a critical interaction with either a significant or coincidental other, finally rendered visible the necessity of professional intervention. Commonly, it was an interaction with a trusted family doctor who detected mental health issues and provided a referral on to specialist services.

*Doctors suggested my responses to their questions were not normal* (59-year-old).

*Sought medical treatment for physical pain, doctor recommended also seeking counselling for underlying depression and anxiety* (27-year-old).

For some men, it was the thought of what would be the repercussions—the “ripple effect”—of their suicide that prompted them to reach out for help. In exhausting the limits of a maladaptive focus on their faults that came with acute suicidality, these men were clearly averse to the thought of their family and friends having to grieve their death, while equally distressing were the practicalities and impact of being found by someone. Many respondents commented on a need to seek help which represented a manifestation of their drive to live up to provider standards entrenched by masculinity socialisation.

I want and need to be here for my son and family. Which made me realize I need to be here for *me* (42-year-old).

*The thought of some random having to deal with my dismemberment and picking me up off the road after I rode my motorcycle into a lamp post* (55-year-old).

As friends, family or employers became aware of the severity of the men’s suffering, sometimes pre-crisis with a pre-emptive push for them to seek help and for others following a breakdown or suicide attempt, these men reached out for formal support to appease or unburden loved ones.

*After various suicide attempts, and constant depression, one of my best friends convinced me that I should try to talk to someone about it and I figured that I had nothing to lose in trying* (22-year-old).

*Eventually I attempted suicide, and after this confided in my sister who convinced me to see a psychologist* (27-year-old).

In a stark example of the power of the “other,” some men reflected on having their lives saved by someone who entered the fundamentally personal space that is a suicide attempt and gave them a chance to seek help and stay alive and well.

*Attempted to commit suicide by hanging, was interrupted in the actions by neighbour barging in and asking questions about what I was up to* (49-year-old).

*Tried to take my own life, wife found me, saved me, and her and my brother helped me to decide on seeing a doctor then a counselor* (55-year-old).

These men pointed to the notion that their own suicidality had a “ripple effect” on those around them, and while it often felt burdensome having others witness and intervene in this suffering, their road to recovery often required someone alongside to guide them.

## 4 Discussion

Whilst our understanding of the intersection between masculinities and suicidality has progressed substantially in recent years, the underlying factors driving men’s help-seeking have gained limited attention. In offering a large group of help-seeking men an opportunity to describe their experiences leading up to this decision to reach out for formal support, this study sought to uniquely explore the intertwining nature of pathways to despair, and those to survival. Often, *Early marginalising traumas,* described in theme one, set the scene for many years of psychosocial adversity by men, exacerbated by often maladaptive coping strategies that represented attempts to retain a sense of masculine control. These men, many of them relaying feelings of marginalisation following their childhood maltreatment, were the most likely to offer detailed, often lengthy accounts of their descent into suicidality and subsequent help-seeking. Comparatively, responses central to theme two, *Cumulative and cascading losses*, reported little history of ongoing distress but rather what appeared to be a swift, even impulsive cascading path to suicidality following the experience of an emasculating psychosocial event, such as the loss of employment, relationship breakdown, or financial distress. The third theme, *Epiphanies on the limits of self-reliance and need for care*, rendered visible the extent to which masculine self-reliance often outweighs desires for professional help-seeking in the context of suicidality. Many men’s suicidality was characterised by lengthy attempts at self-management of symptoms, often through distress concealment and substance use, before reaching a critical inflection point where the necessity to seek help became unavoidable, and for these men, outweighed their belief they were better off dead. Often the visibility of this previously incomprehensible path to support manifested out of sheer hope for some psychological relief. In the final theme, *The circuit breaking role of the “other” in men’s help-seeking*, the (in)direct influence of others—be they doctors, family members, or chance encounters with members of the public—proved pivotal in helping men escape their restrictive, subjective frame and finally decide to seek help. The role of caring masculinities (Elliott, 2015) was clear here too, where many men expressed the commencement of a help-seeking pathway in order to better themselves, for the ultimate betterment of those around them. Each theme and its implications for our understanding of the intersection between masculinities and suicidality is discussed in turn below.

Findings highlighted the role of early emasculating and marginalising traumas including physical, verbal and sexual abuse, that often serve to catalyse and then exacerbate the impact of a suite of aversive psychosocial experiences across the lifespan. This accords broadly with previous research noting the role of early (often childhood) maltreatment and trauma in suicidality (Pompili et al., 2014; Pelletier and Knox, 2017), specifically among men (Rice et al., 2018; Rice et al., 2019). The richly detailed narrative accounts presented by many men in their open text responses broadly reflected a “life-course” perspective on suicidality (Blackman, 2012), which holds that a host of events across the lifespan can coalesce to lead to an individual embodying suicidality as an aspect of their identity, after many years living a life defined by chaos and suffering (Gordon, 2008). This perspective has also been noted in previous research exploring the experiences of suicidality among Canadian gay, bisexual and two-spirit men (Ferlatte et al., 2019). The commonly touted masculine script of “suffering in silence” (Mahalik et al., 2003) was further entrenched by the shame and stigma aligned with early experiences of trauma leading to long delays in help-seeking. This life-history account of undisclosed suicidality is mirrored in existing research where men with a history of sexual abuse delayed help-seeking by an average of 21 years (Easton, 2013).

The restrictive nature of rigid stoicism and toughness in shutting down lines for honest, authentic emotional communication, appeared to render many men unaware of and unable to reflect on the extent and/or abnormality of their suffering. Indeed, many of these men appeared initially unattuned to the role of a critical early trauma in their psychological pain until crisis point, when a “realisation” allowed them to finally confront their trauma. Some men appeared to avoid disclosing their distress due to lacking trust in, or fearing rebuke from others. For other respondents, they simply did not have an outlet or the language to verbalise their suffering. These ideas are present in the broader literature appraising the conditions under which suicidal thoughts arise. A recent study by Nguyen et al. (2021) identified that even when individuals are connected to others, a lack of perceived availability of sources of help can be a driving force in the proliferation of suicidal thinking. Perhaps for some of the men in this study, the marginalisation experienced early in their development, coupled with masculine socialisation, entrenched the idea that they were fundamentally alone in their experience and all adversities could only be handled as such. In sum, consistent in many of these accounts was the notion of a dam of distress that inevitably had to break; an inflection point that moved them from long-term reticence to seeking help, to help-seeking becoming a necessity.

In terms of their reasoning for help-seeking, the extensive narratives provided here could reflect a self-affirming means of reinforcing the rationale behind their help-seeking; that is, in choosing to detail the extent of their suffering, perhaps many respondents were able to feel a sense of justification for their help-seeking and reinforce the hard work required to overcome these traumas. As Mulkey (2004) suggests, men who experience early sexual trauma often encounter an ongoing fragmenting of their masculinity, leading them to seek means to redress this balance by proving and performing their manhood in other ways. The current findings reinforce previous research in suggesting that exploring help-seeking pathways can enable this enactment of masculinity for some men, whereby gaining professional support is recast as a rational means to regain status and control (Oliffe et al., 2012).

Long-term layering of stress atop early traumas described above, can be contrasted with citation of the ostensibly causal role of situational stressors in some men’s largely impulsive, in-the-moment suicidal acts described in theme two. These transpiring situations that triggered distress were commonly tied to traditional ideals of manhood. These findings mirror previous research espousing the pivotal role of situational stressors as driving factors in men’s suicidality (Qin et al., 2000). The most common narratives referenced by respondents were job loss and the ensuing financial difficulties. These precipitants can be linked with a failure to adhere to the provider/breadwinner masculine trope (Shiner et al., 2009; Scourfield et al., 2012). The role of relationship breakdown was also commonly cited, which may reflect the influence of men’s gender socialisation where living up to the responsibility for providing for one’s partner is thought to be indicative of “real” masculinity (Knizek and Hjelmeland, 2018). Relationship deterioration can therefore be perceived by more traditionally-masculine identifying men as a failure to live up to this standard, therefore catalysing a downward spiral in mental illness (Scourfield and Evans, 2015). Additionally, intimate relationship breakdown is thought to pose particular risk for suicidality in men. Many men’s restrictive gender socialisation results in them solely relying on their intimate partner for social and emotional support, in the absence of supportive male friendships (McKenzie et al., 2018). The commonality of these situational stressors in men’s narratives of suicidality also aligns with the literature documenting the role of social isolation in the onset of suicidality (Van Orden et al., 2010; Nguyen et al., 2021). Perhaps an occupationally-derived sense of purpose and work-related social connection represent primary sources of social connection for many men, and the sudden absence of these can catalyse a descent into suicidality.

Building on this, the current data reinforces that it is the transition points as men segue into these demographic groups (i.e., divorcees, retirees) that pose the greatest risk and remain important moments for cross-functional services (e.g., welfare; family courts; health) to assess for distress and actively promote help-seeking. As Krysinska (2014) notes, men may have a unique vulnerability to negative life events and Scourfield and Evans (2015) linked this to role inflexibility and a desire for control over interpersonal outcomes. The vulnerability to these stressors is of particular concern in the male population given the role of risk taking and swift decision making, which narrows the time taken from initial stressors to suicidal thoughts, intent and action (Schrijvers et al., 2012). The intersection with masculinities here may help explain this quick downward trajectory, with the overbearing sense of failure echoed by many men linked to a masculine socialisation which instils a fear-based narrative, where masculine status is precarious (“hard won and easily lost”; Vandello and Bosson, 2013, p. 101) and imbued with a distinct and seemingly unassailable dichotomy between success and failure. Additionally, the role of perceived inaccessibility of sources of help in the onset of suicidal thinking has recently been documented (Nguyen et al., 2021). Socialised self-reliance and independent decision-making may exacerbate the extent to which men in distress feel no help is available, leading to further deterioration of their mental health. For practitioners working with suicidal men, these results underscore a critical need to identify ways of establishing healthy coping mechanisms in the context of these life-course transitions. This could entail challenging notions of competitive masculinities (Connell and Messerschmidt, 2005) and other rigid, gendered cognitive distortions around achievement, emotionality and relationships, underpinning a socially comparative sense of failure (Mahalik, 1999).

The third theme simultaneously provided stark evidence of the extent of many men’s prolonged self-reliance and avoidance of help-seeking in the context of suicidality, while also rendering visible how and when some men reach the limits of this isolationist approach to dealing with their distress. Eventually, often out of fear of the alternative, as suicide grew increasingly prominent as an option in their decision-making process, these men saw value in overcoming pre-existing barriers and seeking professional intervention. For many men, maladaptive coping mechanisms such as substance use, social isolation, distress concealment and risk-taking initially stymied help-seeking. These responses were all in service of upholding rigid self-reliant masculinities, and are all factors which have been identified previously in the literature as warning signs for male suicidality (Player et al., 2015; Seidler et al., 2016). Additionally, while not directly mentioned by respondents, prior research among men has noted mistrust of health professionals as common in delaying help-seeking (Pederson and Vogel, 2007; Johnson et al., 2012), underscoring the importance of beneficial client-practitioner relationships once men do seek help (Kealy et al., 2019; Schultz et al., 2021). In comparison to Cleary’s (2005) eponymous qualitative study “death rather than disclosure,” for the current respondents, an equally strict adherence to stoic masculine performativity inevitably gave way to an epiphanous moment where hope for change arose, often out of desperation and exhaustion. After long periods of often destructive attempts at self-sufficiency, it took a realisation for many respondents that they had reached “rock bottom” that help-seeking arose as a means to mitigate their worsening circumstance.

This notion of having “nothing to lose” in seeking help was also found by River (2018), p. 153 and “turning points” in Ridge et al. (2021), p. 246, where it appeared suicidal men will often exhaust all possible means of dealing with their distress before relinquishing masculine capital and fracturing their self-reliant guise. Indeed, the present findings shed light on the cognitions emblematic of an inflection point towards help-seeking for men. Many respondents described a sense of boredom, frustration or fatigue when reflecting on their ongoing distress, alongside recognising help-seeking or death as their only conceivable options. Given many respondents elaborated on their decision to seek help by reporting a desire for self-betterment in the context of this desperation, interventional efforts could leverage this in future. Instead of simply encouraging men to “talk more” and simply access services early as has been critiqued recently (Chandler, 2021), perhaps public health messaging could focus on men’s agency and reflect on, and leverage, the underlying structures evident in their narratives here. It is perhaps about investigating what structures, from the family to workplace, make narratives like “rock bottom”, “exhausted self-reliance” and “protection of others” possible, and therefore the goal of public health promotion could be to find opportunities to get upstream of these, where men are. A question that remains is when and how protective factors that can underpin resilient responses to distress, like self-reliance, tip into a rigid response pattern, becoming risk factors for certain men and making them more vulnerable to suicide (Krysinska, 2014; Canetto, 2017).

The final theme provides insight into factors that can precipitate decisions to seek help among suicidal men. At various stages in the intertwined paths to suicidality and help-seeking for these men, it was clear that influences of doctors, partners, friends, colleagues and other supports proved instrumental in precipitating the direction to help-seeking. This idea has been discussed in previous research by Vogel et al. (2007) where men’s social networks are often influential in their help-seeking. Oliffe et al. (2021), p. 420 also discussed “saving graces” where men are “saved” by others, be that in directing men to services in the context of serious suicide ideation, or directly thwarting a suicide attempt. This study is nevertheless among the first to highlight that men commonly report the influence of their social networks as the primary driving factor in their help-seeking for suicidality as their internal distress collides with external roles and responsibilities.

The role of the “other” also often took on a more indirect influence, where some men reported the need to stay alive in order to support their loved ones, mirroring the notion that family members can be constructed as “the only anchor” some suicidal men have to the world (Ridge et al., 2021, p. 247). This demonstrates the complexity surrounding caring masculinities among suicidal men; the apparent fracturing of this role in the context of a job loss can at once precipitate suicidal ideation, while simultaneously the drive to care and provide for others can represent a primary reason for help-seeking. Elliott (2015) has previously theorised about the role of caring masculinities in advancing wider societal efforts towards achieving gender equality. The findings presented here add weight to the necessity of public health messaging to leverage this concept in encouraging healthy coping and help-seeking among suicidal men. Past work has shown that a sense of interpersonal burdensomeness and a perceived lack of reciprocal care is common among suicidal individuals (Van Orden et al., 2010). Along these lines, perhaps masculinities that speak to men’s roles as providers can be leveraged for positive effect to communicate to men the value in developing healthy emotion regulation and distress management strategies, such that they can continue to present the best version of themselves to support those around them. This idea has been presented by Scholz et al. (2017), who demonstrated that some men construct help-seeking as responsible and wise, as a means to continue their effective role as a partner, father or worker.

### 4.1 Strengths and Limitations

The present findings shed light on the value of the qualitative survey in researching men’s experiences of help-seeking for suicidality. In the men’s mental health literature there is growing recognition of the importance of considering the contexts within which men are encouraged to “talk” about their experiences of mental ill-health. Chandler (2021) recently critiqued encouragement to “talk about it” as a frequently espoused answer to high rates of suicide in men, where the implication is that if men only opened up more, this would facilitate effective direction to professional services. As Chandler (2021) details, it is critically important to anticipate the consequences of bluntly encouraging men to talk. As such, in allowing men total control over their responses and ensuring anonymity, we argue that this methodology represents a useful avenue to eliciting men’s stories about their suicidality, while also avoiding any undue discomfort for participating men. The choice of methodology here precluded the ability to probe individual participant’s responses relative to comparative qualitative methodologies like interviews or focus groups. We nevertheless contend that the responses obtained here reflect a realistic reflection of the men’s subjective experience, given their largely unguided choice around the length and key content of their responses. As an example, it was telling that many men viewed their help-seeking experience through a medicalised lens, listing nothing more than a cluster of psychiatric diagnoses, whereas others situated their distress in a detailed life history.

Study limitations concern the potentially limited generalisability of these results to non-white populations in Australia, given limited information regarding cultural diversity was obtained beyond whether participants identified as Indigenous. Participating in this study also required internet access and sufficient literacy to comprehend and share one’s story surrounding help-seeking for suicidality; requirements which may have limited the capacity for more disadvantaged men to take part. The findings of this study may also be limited in the extent to which they apply to men seeking help for other reasons than suicidality, such as depression. Nevertheless, in exploring the surrounding literature on men’s qualitative reports of help-seeking for depression, several similarities are noted where men commonly exhaust the limits of self-reliance and approach services in a state of desperation (Johnson et al., 2012). It could therefore be that the themes that were generated here also apply to men’s experiences of help-seeking for depression, albeit potentially earlier in the downward trajectory of distress. Also, whilst a number of assumptions are made about the applicability of the stories examined here to those of men who have died by suicide, it should be acknowledged that there are likely additional factors that may position those who die by suicide as an aetiologically distinct population from the men surveyed here. Nevertheless, many respondents reported at least one suicide attempt, so we maintain confidence in the applicability of these findings to our understanding of suicidality in men, particularly at the point of crisis.

### 4.2 Future Directions

An important area for future research is to explore the temporal aspects of men’s descent into suicidality in greater depth. Previous research has explored the idea that downward distress trajectories and the time from suicidal thoughts to action is shorter among men (Schrijvers et al., 2012). Yet the ways in which oft-cited explanatory factors including masculinities, maladaptive coping and impulsivity feed into this require further research. Investigating this in greater depth may shed light on the ways in which direction to help-seeking, or even the development of insight into more psychosocially adaptive coping methods, can become a more prominent juncture in this pathway. Further research is needed to better understand how factors proposed here (e.g., early trauma; situational stressors) alter the dimensions of the “window of opportunity” for intervention among suicidal men. This study sought to understand the reasons men sought help, but the quality of that care, and men’s satisfaction and outcomes in moments of crisis remain under-explored. A follow-up study extending on Cleary’s (2012) seminal work which explored engagement rates and attitudes towards help-seeking among suicidal men, by undertaking an in-depth review of what does and does not work for this critical demographic in care would greatly benefit the field.

### 4.3 Conclusion

This study has provided a pertinent exploration of the paths to help-seeking for suicidality among men, and sheds light on the important ways that masculinities are negotiated and preserved throughout this process. Given the gendered dimensions of suicidality, it is crucial that future research seeks to embed critical reflection on masculinities in the entire pathway from risk factors to help-seeking in the context of crisis.

## Data Availability

The datasets presented in this article are not readily available because of ethical restrictions. Requests to access the datasets should be directed to zac.seidler@orygen.org.au.
